# Anti-fibrotic Effects of CXCR4-Targeting i-body AD-114 in Preclinical Models of Pulmonary Fibrosis

**DOI:** 10.1038/s41598-018-20811-5

**Published:** 2018-02-16

**Authors:** K. Griffiths, D. M. Habiel, J. Jaffar, U. Binder, W. G. Darby, C. G. Hosking, A. Skerra, G. P. Westall, C. M. Hogaboam, M. Foley

**Affiliations:** 1 AdAlta Limited, La Trobe University, 15/2 Park Drive, Bundoora, 3083 Australia; 2 The Department of Biochemistry and Genetics, La Trobe Institute for Molecular Science, La Trobe University, Bundoora, Melbourne 3086 Australia; 3 Cedars-Sinai, Medical Centre, Los Angeles, CA 90048 USA; 4 Department of Respiratory Medicine, Alfred Hospital and Monash University, Melbourne, Victoria 3000 Australia; 5 XL-protein GmbH, Lise-Meitner-Str. 30, 85354 Freising, Germany

## Abstract

Idiopathic pulmonary fibrosis (IPF) is a chronic fibrotic lung disease that is prevalent in individuals >50 years of age, with a median survival of 3–5 years and limited therapeutic options. The disease is characterized by collagen deposition and remodeling of the lung parenchyma in a process that is thought to be driven by collagen-expressing immune and structural cells. The G-protein coupled C-X-C chemokine receptor 4, CXCR4, is a candidate therapeutic target for IPF owing to its role in the recruitment of CXCR4^+^ fibrocytes from the bone marrow to fibrotic lung tissue and its increased expression levels by structural cells in fibrotic lung tissue. We have engineered a novel fully human single domain antibody “i-body” called AD-114 that binds with high affinity to human CXCR4. We demonstrate here that AD-114 inhibits invasive wound healing and collagen 1 secretion by human IPF fibroblasts but not non-diseased control lung fibroblasts. Furthermore, in a murine bleomycin model of pulmonary fibrosis, AD-114 reduced the accumulation of fibrocytes (CXCR4^+^/Col1^+^/CD45^+^) in fibrotic murine lungs and ameliorated the degree of lung injury. Collectively, these studies demonstrate that AD-114 holds promise as a new biological therapeutic for the treatment of IPF.

## Introduction

Idiopathic Pulmonary Fibrosis (IPF) is the most common Interstitial Lung Disease (ILD), with a poor prognosis and median survival of 3–5 years after diagnosis. IPF is characterized histologically by the pattern of Usual Interstitial Pneumonia (UIP), consisting of fibroblastic foci, which are the site of active tissue remodeling due to the presence of activated fibroblasts and myofibroblasts. Currently, two therapeutics have been approved for the treatment of IPF, pirfenidone^1–6^ and nintedanib^7–10^, both of which have been shown to slow, but not halt disease progression. Thus, there is an unmet clinical need to develop next generation therapeutics with improved clinical efficacy.

The fibrotic triggers in IPF are unknown but it is speculated that persistent lung injury leads to alveolar epithelial cell injury and death, and subsequent aberrant repair mechanism(s) ablates the alveolus^11^. Mechanisms leading to the progression of fibrosis in IPF remain controversial; however various reports suggest that invasion of fibroblasts from fibrotic into normal areas of the lungs^12,13^, and the recruitment of collagen 1-expressing fibrocytes and their differentiation into matrix producing fibroblasts, in a CD44, hyaluronan and β-arrestin-dependent mechanism^12,13^ may play a major role. Additionally, chemokines and chemokine receptors have been shown to promote cellular invasion in inflammation, cancer and fibrosis, via mechanisms involving various adapter molecules and signaling pathways, including CD44, integrins, matrix metalloproteases and β-arrestin^14,15^. The role of chemokines and chemokine receptors in lung remodeling, fibroproliferation and fibrosis has been reviewed^16^. C-X-C chemokine receptor 4 (CXCR4) is an alpha chemokine receptor, known to bind to the C-X-C chemokine, CXCL12. CXCR4 signaling has been observed to play a role in several pathological processes including invasion of pancreatic cancer, Ewing sarcoma, esophageal cancer, gliomas and gastric cancer^17–21^ and promotion of pulmonary^16^ and kidney fibrosis^22^. Indeed, various studies have shown that inhibition of CXCR4 results in anti-fibrotic effects *in vitro* and ameliorated bleomycin induced lung fibrosis *in vivo*^22–26^, suggesting that this chemokine receptor might be a therapeutic target in fibrotic lung diseases.

We have previously described the i-body AD-114, a human single domain antibody that specifically antagonizes CXCR4 and has a different mechanism compared to other CXCR4 antagonists^27^. In this report, we describe the efficacy of AD-114 in modulating mechanisms of lung fibrosis, both *in vitro* and *in vivo*. Immunohistochemical (IHC) analysis showed that CXCR4 is expressed by epithelial, interstitial and immune cells in the lungs of IPF patients, a finding confirmed by flow cytometry. Treatment with AD-114 but not small-molecule CXCR4 antagonist, AMD3100 (plerixifor, currently approved for mobilization of hematopoietic stem cells^28^) reduced IPF lung fibroblast invasive wound healing and collagen 1 secretion, especially in fibroblasts derived from IPF patients showing a slow progressive decline of lung function (as previously defined^29^). In murine models of bleomycin-mediated lung injury and fibrosis^30–32^, AD-114 treatment, especially by applying a half-life extended format^33^, ameliorated lung remodeling as determined by histological, biochemical and transcriptomic quantification of collagen expression and deposition. Specifically, anti-fibrotic effects of AD-114 in the murine bleomycin model appear to be due to reduction of collagen transcript and protein expression and reduced migration of CXCR4^+^Col^+^CD45^+^ fibrocytes to the injured lung. Collectively, our results suggest that utilizing CXCR4 specific i-body AD-114 in IPF patients might reduce disease progression and prove beneficial in treating this disease.

## Results

### Validation of activity of AD-114 binders in various formats

**Figure 1:**
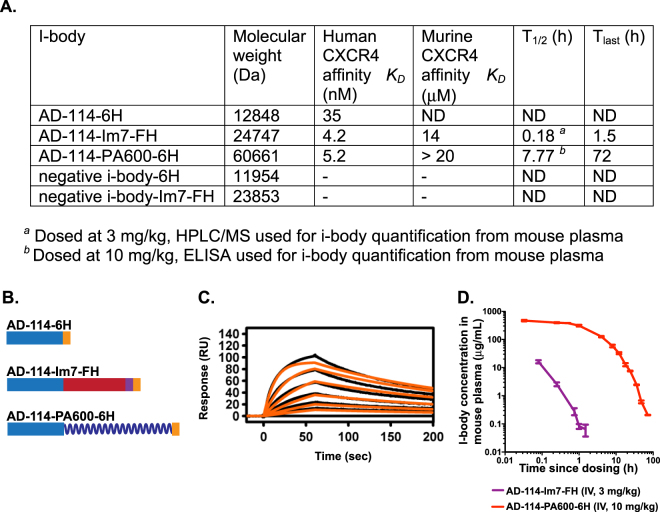
Attributes of AD-114 variants. AD-114 variants were expressed as heterologous proteins in *Escherichia coli* or *Pichia pastoris*. The molecular weight of i-body variants was determined by mass spectrometry, binding kinetics were determined by SPR, T_1/2_ and T_last_ values were obtained from *in* *vivo* murine pharmacokinetic studies by non-compartmental analysis of the mean plasma concentration of various i-bodies, N = 3 mice per group. (**A**). Various conjugates were added at the C-terminus of AD-114 (blue) to improve solubility (Im7, red) and circulating half-life (PA600, zig zag). Purification tags were His_6_ hexapeptide (orange) and FLAG (purple) (**B**). Kinetic data set collected for AD-114-PA600-6H binding to immobilized human CXCR4 lipoparticles. Injected i-body concentrations were 160, 80, 40, 20, 10, 5, and 2.5 nM. Data are shown in black and fits to single site kinetic model with mass transport are shown in orange (**C**). *In vivo* pharmacokinetic data from mice showing decrease in the plasma concentration of various i-bodies over time, N = 3, error bars show S.E. (**D**).

CXCR4-specific i-body AD-114 was expressed in *E. coli* or *P. pastoris* in 3 different C-terminal formats (Fig. 1A,B). The affinity of the different AD-114 formats was determined by SPR using human or murine CXCR4 lipoparticles (Fig. 1A,C). Affinity for human CXCR4 of AD-114 produced in *E. coli* in Im7-FH or PA600-6H formats was *K*_*D*_ = 4.2 and 5.2 nM, respectively, which is consistent with previous findings for AD-114^27^. Although AD-114-6H produced in *P. pastoris* had a lower affinity for human CXCR4, it still bound with a *K*_*D*_ of 35 nM (Fig. 1A). Thus, the i-body was generally tolerant of modification at the C-terminus of the protein. The affinity of AD-114 produced in *E. coli* (as Im7-FH or PA600-6H^33^ formats) for murine CXCR4 was lower but a precise affinity was difficult to determine using SPR, since the murine CXCR4 lipoparticles were not as stable as the human CXCR4 lipoparticles in this format.

### Half-life extension

AD-114 with an Im7 tag was found to have a half-life of only 18 min in mice and the i-body remained in the bloodstream for 1.5 h. In contrast, AD-114-PA600-6H had a dramatically improved half-life in the bloodstream of mice with a T_1/2_ of 7.77 h and a residence time of 72 h. (Fig. 1D). Thus, C-terminal modifications, in particular PASylation^33^, can allow tailoring of the pharmacokinetics properties of AD-114 i-body, which offers benefits for translation into the clinic.

### Expression of CXCR4 in NDC and IPF lung tissue

**Figure 2:**
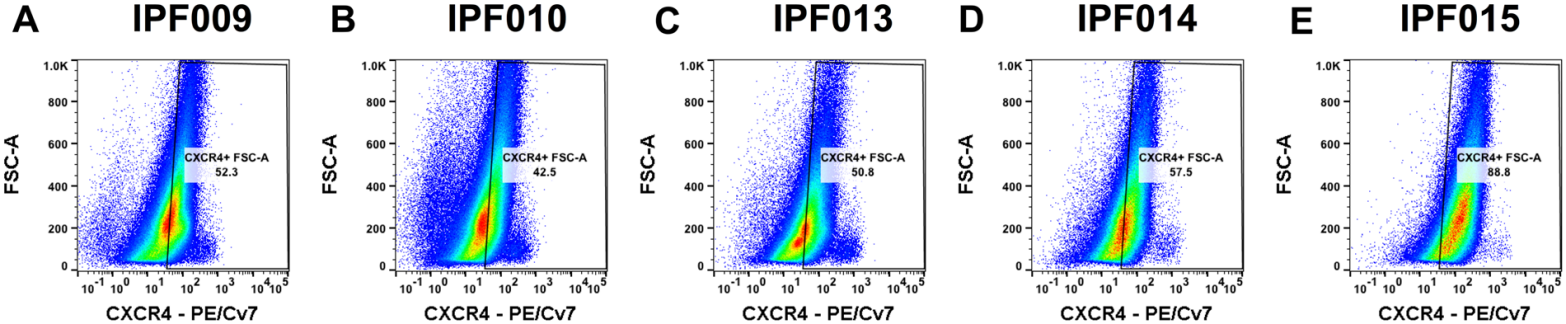
CXCR4 is abundantly expressed by most of end-stage IPF lung explant cells. Cellular suspensions were generated from freshly isolated IPF lung explants, stained with anti-CXCR4 antibody and analyzed by flow cytometry. (**A**–**E**) Depicted are flow cytometric dot plots from 5 IPF lung explants showing cells stained for CXCR4.

**Figure 3:**
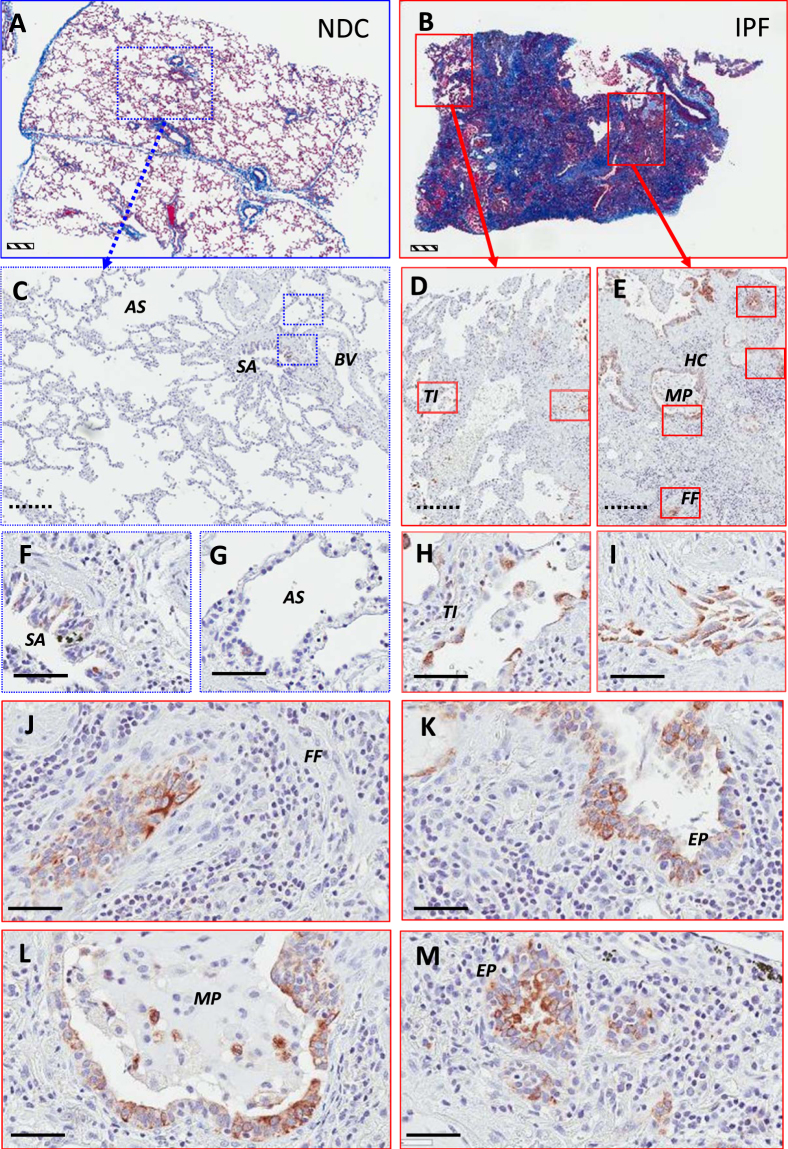
Immunohistochemical staining for CXCR4 in IPF and NDC lung tissue. Immunohistochemical staining of human lung tissue from a patient with IPF (**B**,**D**,**E**,**H**–**M**) and a NDC donor without lung disease (**A**,**C**,**F**,**G**). Masson’s trichrome staining for collagen (blue) was used to assess extent of fibrosis (**A**,**B**). CXCR4 positivity was identified using a commercially available, validated antibody and visualized using DAB (3,3′-diaminobenzidine) (brown). Tissue was counterstained in Mayer’s hematoxylin. (**A**) Masson’s trichrome staining of NDC lung tissue section. (**B**) Masson’s trichrome staining of a IPF lung tissue section. (**C**) CXCR4 is largely absent in the NDC lung tissue. (**D**) CXCR4^+^ cells can be seen in less fibrotic areas of lung tissue. (**E**) In densely fibrotic regions of lung tissue, CXCR4^+^ cells can be seen in the hyperplastic epithelium (EP) of honeycomb cysts (HC), within a mucus plug (MP), and immediately adjacent to fibroblastic foci (FF). (**F**) At higher magnification, some CXCR4^+^ cells are seen within the NDC small airway (SA). (**G**) Very few, if any, CXCR4^+^ cells are seen in the NDC alveolar interstitium and within the alveolar space (AS). (H) CXCR4^+^ cells can be seen lining thickened interstitium (TI). (**I**) CXCR4^+^ cells are also seen within fibrotic interstitial tissue. (**J**) Adjacent to FF, CXCR4^+^ cells are seen as a dense grouping far away from any identifiable airway. (**K**) CXCR4+ cells appear epithelial and are present in the damaged airway. (**L**) Additionally, in areas of dense fibrosis where the formation of HC are apparent, cells that are CXCR4^+^ appear more cuboidal shaped and are lining the edges of the cyst. (**M**) Fibrotic airway with CXCR4^+^ cells with an epithelial appearance. Scale bars: striped (500 μm); dotted (200 μm); solid (60 μm). AS alveolar space, BV blood vessel, EP hyperplastic epithelium, FF fibroblastic foci, HC honeycomb cyst, MP mucus plug, TI thickened interstitium, SA small airway.

Flow cytometric analysis of cells dissociated from explanted IPF lung tissues showed that CXCR4 is abundantly expressed by cells from end-stage fibrotic lungs **(**Fig. 2A–E). Immunohistochemical (IHC) analysis of IPF tissue using a commercial anti-CXCR4 antibody showed expression of CXCR4 in human lung tissue from IPF patients with little staining in lung tissue from age-matched non-disease controls (NDC; Figs 3 and S1). Specific staining in IPF lung tissues was apparent in areas of dense fibrosis (as assessed by Masson’s trichrome and IHC staining on serial sections; Fig. 3B,E), where staining was observed in hyperplastic epithelial cells localized in airways, honeycomb-cysts and adjacent to fibroblastic foci (Fig. 3E,H,J–M). Further, staining was also observed on cells localized in the fibrotic interstitial tissue (Fig. 3I). These results suggest that CXCR4 is abundantly expressed in IPF lung tissues.

**Figure 4:**
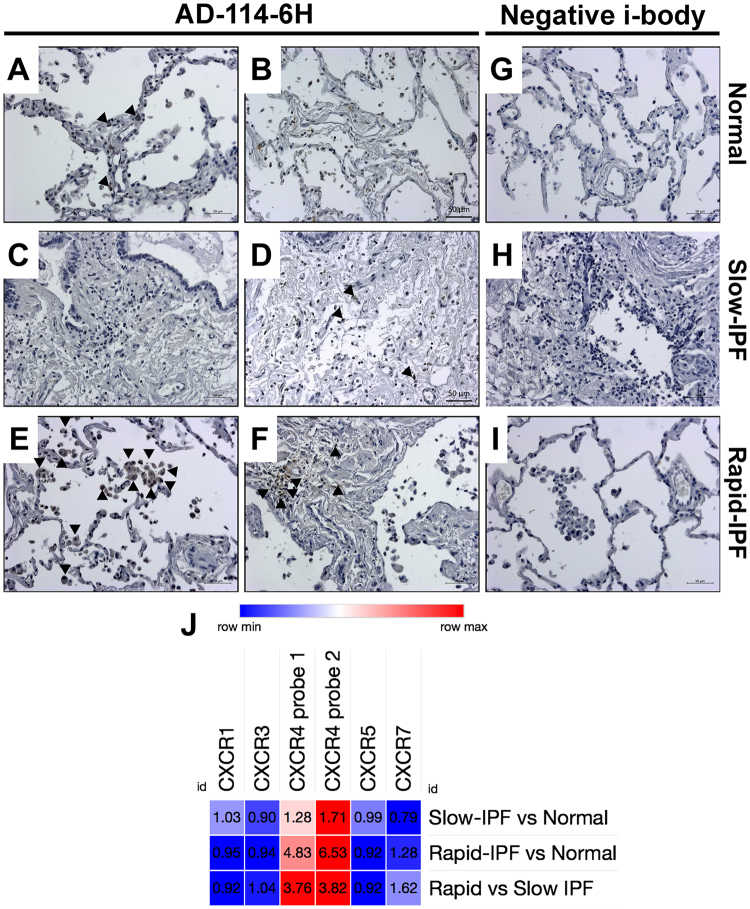
CXCR4 expression in fibroblasts. (**A**–**I**) NDC lung explants (**A**,**B** and **G**), slow- (**C**,**D** and **H**) and rapid- (**E**,**F** and **I**) IPF lung biopsies were stained with CXCR4 specific i-body (AD-114; **A**–**F**) and negative i-body (**G**–**I**). Black arrowheads mark cells that are expressing CXCR4 and stained by AD-114-6H. Scale bars are 50 μm. (**J**) Publicly available gene expression arrays of lung fibroblasts derived from diagnostic IPF lung biopsies from slow- or rapid-progressing IPF patients or NDC lung explants (GSE44723) were analyzed using NCBI’s Geo2R as follows: Slow-IPF versus NDC lung fibroblasts, Rapid-IPF vs NDC lung fibroblasts or rapid- versus slow-IPF. Depicted is a heat map showing increased (red) or decreased (blue) expression of C-X-C chemokine receptors by lung fibroblasts. The average fold change of expression (as determined for each array probe corresponding to the C-X-C chemokine receptor) is depicted in the heat maps. No statistically significant expression changes were observed.

To assess the binding of AD-114-6H to lung tissue associated CXCR4, IHC staining with AD-114-6H was initially optimized on spleen and liver tissue from immunocompromised SCID/bg mice that had been challenged with an intravenous injection of CXCR4-expressing human leukemic T cells, CCRF-CEM cells, (^27,34–36^) **(**Figure S2**)**. AD-114 immunolabeled few cells in NDC lung explants (Fig. 4A,B) and slow-IPF lung biopsies (Fig. 4C,D). However, this i-body immunolabeled many immune and interstitial (Fig. 4E,F) cells in rapid-IPF lung biopsies, suggesting high CXCR4 expression in rapid-IPF lungs. Negative i-body did not label any cells in normal, slow-IPF or rapid-IPF lung tissues (Fig. 4G–I). Finally, to assess the expression of CXCR4 in lung fibroblasts, publicly available gene expression arrays (GSE44273) were mined for C-X-C transcript expression in slow-IPF, rapid-IPF (as previously defined^29^) and NDC lung fibroblasts. CXCR4 was the most up-regulated C-X-C chemokine receptor in both slow- and rapid-IPF relative to NDC lung fibroblasts, where this chemokine receptor was more abundantly expressed in rapid-IPF relative to slow-IPF and normal lung fibroblasts (Fig. 4J). Collectively these results suggest that CXCR4 is expressed by immune and structural cells in IPF lung tissue.

### AD-114 modulates IPF lung fibroblast invasion and collagen 1 secretion

A role for CXCL12 and CXCR4 in invasion has been shown in several reports^17–22^. Thus, to determine the role of CXCR4 and CXCL12 in fibroblast invasion, NDC and IPF lung fibroblasts were utilized in an invasive wound healing assay. AD-114 was tested alongside the small-molecule CXCR4 antagonist, AMD3100 (plerixifor). AD-114 had no effect on motility of fibroblasts from two NDC donors (Figure S3A–B), but effectively (and more consistently relative to AMD3100) inhibited fibroblast motility from three IPF patients (Figure S3C–E). The negative i-body had minimal effect on invasion of all fibroblasts with the exception of S98 (Figure S3D).

**Figure 5:**
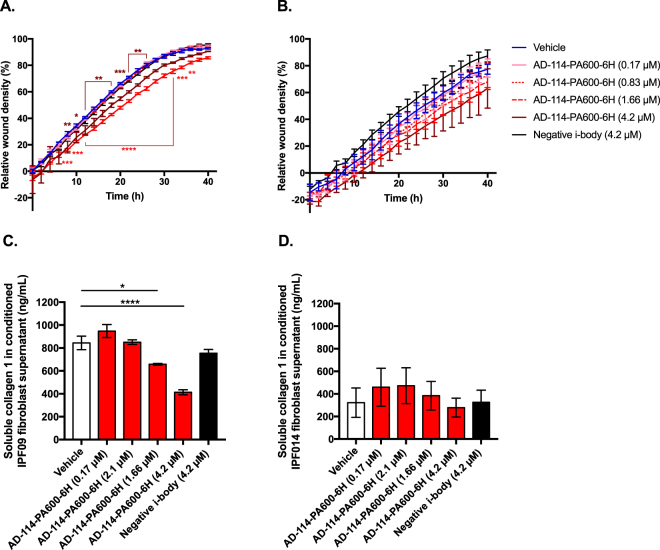
AD-114-PA600-6H reduces migration and collagen production in fibroblast cell lines. Lung fibroblasts from IPF patients (**A**) IPF09 (slow-IPF) and (**B**) IPF14 (rapid-IPF) were plated onto BME coated wells, scratched and then layered with 2 mg/mL BME containing AD-114-PA600-6H or negative i-body. Invasion was measured for 40 h following treatment. N = 3, error bars show S.E. Statistical significance if any is shown as *p ≤ 0.05 **p ≤ 0.01 ***p ≤ 0.001 ****p ≤ 0.0001 as determined by comparing the Vehicle mean against treated mean using a Two-way ANOVA multi-comparison test, Tukey’s posthoc test. Top and bottom significance values indicate significance for cells treated with 1.66 and 4.2 µM AD-114-PA600-6H, respectively. The error bars were larger in the latter group possibly due to active senescence pathways in these cells. AD114-PA600-6H also reduced collagen protein in IPF lung fibroblasts from IPF-09 but not IPF-014 (**C**,**D**) in conditioned supernatants of fibroblast 72 h post treatment with the i-body. N = 3, error bars show S.E. Statistical significance if any is shown as *p = < 0.05 or ****p = < 0.0001 as determined following testing of Vehicle mean against treatment means using a One-way ANOVA, Dunnett’s posthoc test.

The potential contribution of CXCR4 to lung fibroblast activation and extracellular matrix generation was explored by testing the efficacy of AD-114 with the half-life extension conjugate PA600-6H to modulate IPF lung fibroblast invasion and collagen 1 protein secretion. AD-114-PA600-6H effectively inhibited invasive wound healing of two IPF lung explant derived fibroblasts, IPF09 and IPF014, at ≥1.66 µM and 0.83 µM, respectively (Fig. 5A,B). Further, this i-body significantly inhibited (in a dose dependent manner) collagen 1 protein secretion in IPF09 (Fig. 5C) but not IPF014 (Fig. 5D), relative to negative i-body control. These results suggest that CXCR4 activation might promote IPF fibroblast invasion and extracellular matrix protein secretion.

### Effect of AD-114 in bleomycin models of lung injury

**Figure 6:**
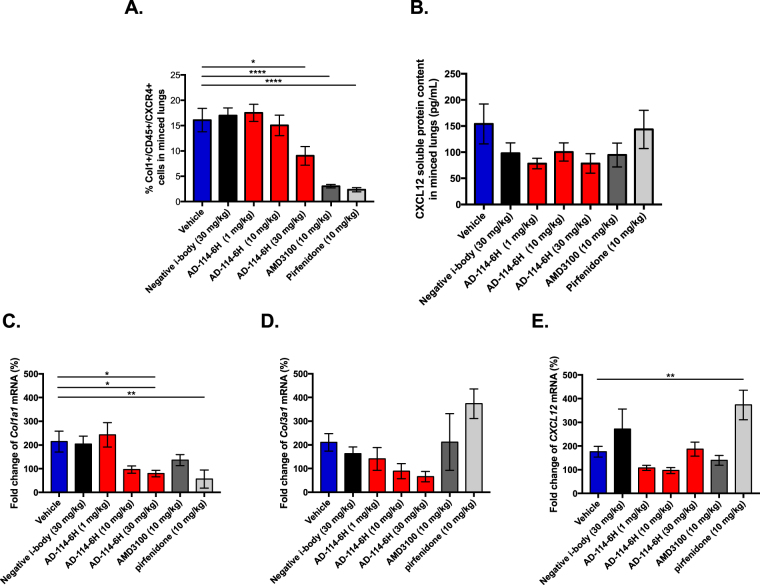
In a 4-day murine bleomycin injury model in therapeutic treatment mode, AD-114-6H reduced the % of Col1^+^/CD45^+^/CXCR4^+^ cells and CXCL12 content in the lungs, and modulated the expression of *col1a1*, *col3a1* and *cxcl12* genes. Following intratracheal instillation of bleomycin, mice were injected daily for 4 days with vehicle, AD-114-6H, negative i-body, AMD3100 or pirfenidone. Cell suspensions from minced lung tissue were evaluated by flow cytometry for Col1^+^/CD45^+^/CXCR4^+^ cell content (**A**) and by qPCR for expression of genes encoding *col1a1*, *col3a1* and *cxcl12* (**C**–**E**), and the CXCL12 soluble protein content in the lung bronchoalveolar lavage (BAL) fluid was quantified by ELISA (**B**). N = 10 mice per group, error bars show S.E. Statistical significance if any is shown as *p = < 0.05, **p = < 0.01 or ****p = < 0.0001 as determined following testing of Vehicle mean against treatment means using a One-way ANOVA, Dunnett’s posthoc test.

To determine the efficacy of AD-114 in modulating pulmonary fibrosis *in viv*o, mice were challenged with 2U/kg of bleomycin followed by daily prophylactic treatment of AD-114-6H (dosed at 1, 10 and 30 mg/kg), negative i-body (dosed at 30 mg/kg), AMD3100 or pirfenidone (both dosed at 10 mg/kg). After 4 days of bleomycin administration, fibrocytes (defined as CXCR4^+^Col^+^CD45^+^) made up approximately 15% of all cells in the bleomycin-challenged and vehicle or negative i-body treated mice; however, AD-114-6H at 10 and 30 mg/kg reduced the percentage of fibrocytes in a dose dependent manner, down to 9% at 30 mg/kg (Fig. 6A). AMD3100 and pirfenidone reduced fibrocyte content to 3 and 2%, respectively (Fig. 6A). Treatment with AD-114-6H correlated with a trending reduction in CXCL12 protein levels in the bronchoalveolar lavage (BAL), especially at 1 and 30 mg/kg, compared to the vehicle only treatment; however, the negative i-body and AMD3100, but not pirfenidone, also reduced CXCL12 content by approximately 33% (Fig. 6B). AD-114-6H consistently reduced *col1a1*, *col3a1* and *cxcl12* transcripts, in the lungs of bleomycin challenged mice; whereas AMD3100 and pirfenidone reduced *col1a1* but not *col3a1* or *cxcl12* transcripts, 4 days after bleomycin administration (Fig. 6C–E).

**Figure 7:**
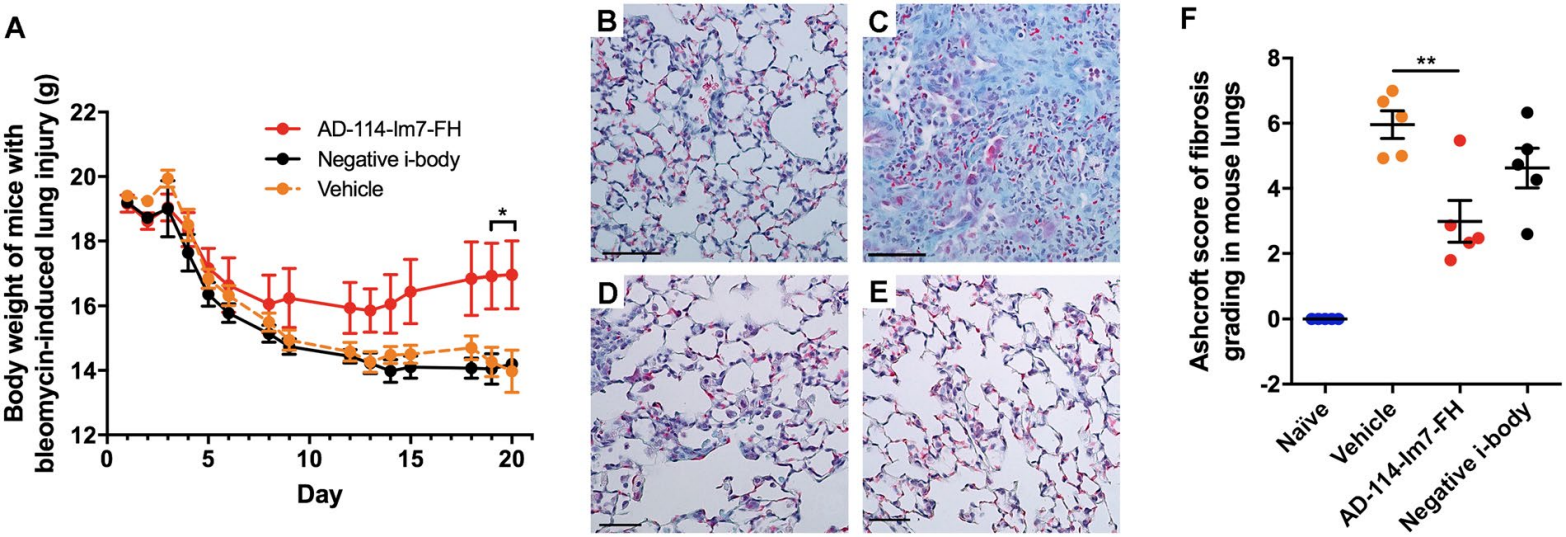
Bleomycin-induced lung injury in mice is ameliorated by treatment with AD-114-Im7-FH. Mice were injected daily for 21 days with vehicle, AD-114-Im7-FH, negative i-body or AMD3100 at 10 mg/kg. (**A**) Body weights of each group were evaluated and are depicted. (**B**–**E**) Masson’s trichrome staining of lung histological samples from untreated healthy mice (**B**), mice 21 days after treatment with bleomycin (**C**) mice treated with bleomycin and then AD-114-Im7-FH (**D**) or AMD3100 (**E**) for 21 days. Scale bars represent 50 μm. (**F**) The degree of lung fibrosis was scored according to the scale defined by Ashcroft *et al*.^37^. N = 5 mice per group, error bars show S.E. In A, statistical significance is shown as *p = < 0.05, as determined following testing of the Vehicle against AD-114-Im7-FH using a Two-way ANOVA multicomparison test, Tukey’s posthoc test. In F, statistical significance if any is shown as **p = < 0.01 as determined following testing of Vehicle mean against treatment means using a One-way ANOVA, Dunnett’s posthoc test.

**Figure 8:**
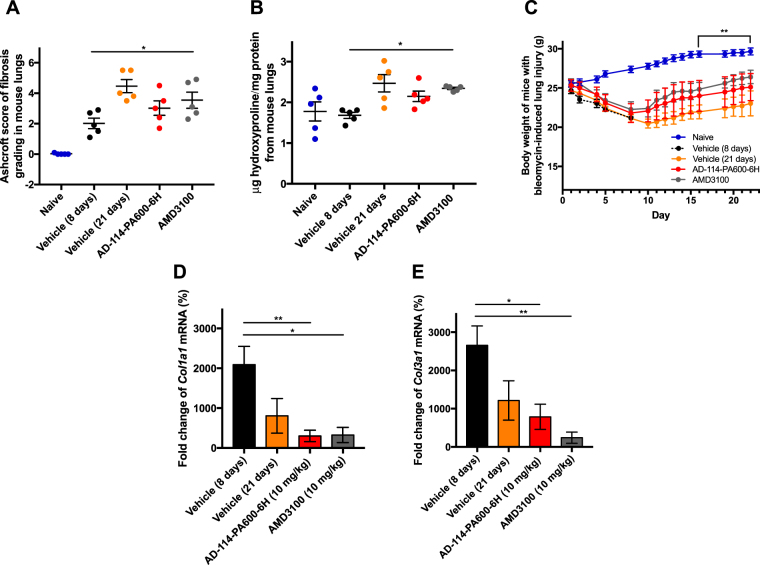
In a 21-day therapeutic model AD-114-PA600-6H protected mice from the development of pulmonary fibrosis, reduced hydroxyproline (collagen) content, reduced weight loss associated with bleomycin injury and decreased expression of *col1a1* and *col3a1* genes. Mice were intratracheally instilled with bleomycin then injected daily with vehicle, or from t = 8–21 days with AD-114-PA600-6H or AMD3100 at 10 mg/kg. Depicted are (**A**) degree of fibrosis according to the Ashcroft scale^37^, (**B**) lung hydroxyproline content, (**C**) body weights and expression of (**D**) *Col1a1* and (**E**) *Col3a1* genes. N = 5 mice per group, error bars show S.E. In C, statistical significance is shown as **p = < 0.01 as determined following testing of the Vehicle (21 days) against AD-114-PA600-6H using a Two-way ANOVA multicomparison test, Tukey’s posthoc test. In D and E, statistical significance if any is shown as *p = < 0.05, **p = < 0.01 as determined following testing of the Vehicle (8 days) mean against treatment means using a One-way ANOVA, Dunnett’s posthoc test.

To further assess the efficacy of AD-114-Im7-FH in modulating bleomycin induced lung fibrosis, mice were challenged with saline or bleomycin followed by daily prophylactic treatment with i-body or small molecule inhibitors for 21 days. Body weight measurements showed that mice treated with AD-114-Im7-FH had the least overall weight reduction relative to vehicle and negative i-body treated groups after bleomycin administration (Fig. 7A). Masson’s trichrome staining (Fig. 7B–E) and Ashcroft scoring^37^ (Fig. 7F) of fibrotic mouse lungs showed that prophylactic treatment with AD-114-Im7-FH (Fig. 7C,E) or AMD3100 (Fig. 7D) markedly ameliorated fibrotic lung remodeling relative to vehicle + bleomycin treated groups (Fig. 7B,E). Naïve mice which did not receive bleomycin showed no signs of abnormal lung structure (Fig. 7A) and scored 0 on the Ashcroft scale (Fig. 7E). To determine the therapeutic potential of targeting CXCR4 in this preclinical model of lung fibrosis, mice were treated starting at day 8 after bleomycin administration, at which point daily dosing commenced with either vehicle, AD-114-PA600-6H or AMD3100 (both at 10 mg/kg) until day 21. Bleomycin administration induced lung remodeling as early as day 8 and apparent remodeling after 21 days, as evident by histological Ashcroft scoring of vehicle treated groups (Fig. 8A). Daily administration of AD-114-PA600-6H from day 8 to day 21 markedly ameliorated fibrotic lung remodeling relative to the vehicle treated group, as assessed by Ashcroft Scoring (Fig. 8A). Further, AD-114-PA600-6H treated groups at day 21 showed similar Ashcroft Scores to the lungs of vehicle treated groups, 8 days after bleomycin administration (Fig. 8A), suggesting that the progression of lung fibrosis was ameliorated after the commencement of AD-114-PA600-6H administration. However, AMD3100 also reduced the Ashcroft score, but to a lesser extent relative to AD-114-PA600-6H, since there was a statistically significant difference between the AMD3100 treated group and the vehicle treated group at day 8 (Fig. 8A). Biochemical hydroxyproline (a major component of collagen^38^) quantification supported the Ashcroft scoring results, where there was a significant elevation of hydroxyproline content in vehicle + bleomycin treated and a trending reduction in the AD-114-PA600-6H + bleomycin and AMD3100 + bleomycin treated groups (Fig. 8B). Further, when compared to the vehicle control, therapeutic administration of AD-114-PA600-6H or AMD3100 ameliorated body weight loss approximately 16 days after bleomycin administration (Fig. 8C). Finally, transcriptomic analysis showed that both AD-114-PA600-6H and AMD3100 treatment induced a modest reduction in the expression of *col1a1* and *col3a1* transcripts (Fig. 8D–E). Collectively, these results suggest that targeting CXCR4 signaling via AD-114-PA600-6H or AMD3100 effectively ameliorated fibrotic lung remodeling in a pre-clinical murine model of lung fibrosis.

## Discussion and Future Direction

IPF is a progressive and typically lethal fibrotic lung disease, characterized by the presence of fibroblast rich foci containing cells actively remodeling the lung, ultimately leading to tissue destruction and loss of lung function. IPF has been observed to progress at different rates. Some IPF patients show no or slow loss of lung function while others show rapid deterioration and loss of lung function^39^. Mechanisms contributing to the progression of fibrosis in IPF remain elusive, however, various reports suggest that fibroblast invasion might play a major role in the spread of this disease^12,13^. Currently, there is no consensus on pathways promoting cellular invasion, yet, several studies have implicated the CXCR4/SDF-1 axis in development of fibrosis and one report suggests an important role for β-arrestin pathways in lung fibroblast invasion and pulmonary fibrosis^12^. A profibrotic role for this chemokine receptor has been previously demonstrated in the literature, where CXCR4 signaling is thought to mediate the recruitment of CXCR4^+^ fibrocytes to the lungs^23–26,40^. Additionally, studies have shown that CXCR4 has pro-proliferative and pro-survival roles in various normal and tumor cells through mechanisms involving activation of the Akt pathway^41–44^ and that this chemokine receptor may promote myofibroblast differentiation in the prostate^45^. Further, Li *et al*. have shown a role for CXCR4 in promoting epithelial to mesenchymal transition of pancreatic cancer cells through the activation of the non-canonical hedgehog signaling pathway, suggesting that there may be alternative “non-canonical” signaling mechanisms utilized by this chemokine receptor to promote fibrosis^17^.

In this report, we have demonstrated a role of CXCR4 in the promotion of fibrosis using *in vitro* and *in vivo* approaches. IHC analysis shows that CXCR4 is primarily expressed by lung epithelial cells in IPF lung tissues, but is also expressed by immune cells and cells localized in the fibrotic interstitium. The role of this chemokine receptor on epithelial cell function remains elusive and warrants future investigation. Transcripts for this chemokine receptor were markedly elevated in IPF relative to NDC lung fibroblasts, especially in fibroblasts derived from rapid-progressing IPF patients’ lung biopsies. Further, *in vitro* studies suggested that CXCR4 signaling in lung fibroblasts promoted cell invasion and the secretion of collagen 1, and blockade of this receptor using the i-body AD-114 inhibited both invasive wound healing and collagen 1 secretion. Interestingly, the inhibitory effect of AD-114 was more apparent in slow-progressing IPF lung fibroblasts relative to rapidly progressing-IPF lung fibroblasts, potentially due to the more prominent senescence phenotype of the latter, as assessed via transcriptomic analysis for senescence associated markers, CDKN1A and NOX4 and elevated ß-galactosidase activity (Figure S4). Indeed, fibroblasts showing a more prominent senescence phenotype invaded less, relative to proliferating fibroblasts. Finally, in our studies, AMD3100 was not effective in inhibiting fibroblast invasion, potentially due to its known agonistic effect for CXCR7^46^. We propose that rather than simply blocking the interaction between CXCR4 and its ligand CXCL12, AD-114 can bind to CXCR4 and modulate intracellular signalling. We have previously shown that AD-114 can modulate β-arrestin signaling by CXCR4^27^. Given the known role of ß-arrestin signaling in lung fibroblast invasion^12^ and the activation of this pathway by CXCR4, through CXCR7 signaling^40,47,48^, it is likely that AD-114, but not AMD3100, might modulate lung fibroblast invasion via the inhibition of CXCR4 mediated activation of ß-arrestin. These results suggest that targeting CXCR4 might effectively inhibit lung fibroblast invasion and collagen 1 secretion in diseased fibrotic lungs.

Increased migration of fibrocytes or a similar circulating progenitor cell type to the diseased fibrotic lung has been reported to correlate with poor prognosis in IPF^16,49^, although it is not clear how these cells contribute to fibrosis. Several studies utilizing mouse models of lung fibrosis have reported positive results after effective targeting of the CXCR4-CXCL12 axis with reagents such as the small molecule CXCR4 antagonist AMD3100^23,24^ or a neutralizing anti-CXCL12 antibody^50^. Efficacy in these studies was reported to be potentially due to reduced trafficking of fibrocytes to the lungs, reduced collagen deposition and overall fibrosis by Ashcroft scoring, histological staining and/or biochemical quantification of hydroxyproline. Despite weaker binding to mouse relative to human CXCR4 (as assessed on murine CXCR4 lipoparticles), AD-114 ameliorated symptoms of fibrosis in the murine bleomycin studies as evidenced by reduced degree fibrosis (as assessed by Ashcroft scoring) and reduced bleomycin mediated loss of body weight. These observed beneficial effects, elaborated by AD-114 administration, correlated with reduced fibrocyte migration to the lung tissue, which is consistent with AD-114 inhibiting the innate chemotactic function of CXCR4. Previous studies in a murine air-pouch model also showed an inhibitory effect of AD-114 on cell migration^27^. This might be due to the binding of  the i-body to four out of six critical residues in human CXCR4 (previously identified by epitope mapping to be bound by AD-114 (W195, D262, L266, E288)) that are conserved in the murine CXCR4 sequence. In addition to its inhibitory effect on CXCR4-mediated migration, AD-114 appeared to reduce transcript and protein levels of *Col1a*. Given the modulation of lung fibroblast invasion and collagen secretion by AD-114 *in vitro*, it is possible that some of the anti-fibrotic effects observed with this i-body *in vivo* might be due to its anti-fibrotic effects on mouse resident lung fibroblasts. The efficacy of AD-114 in murine models of pulmonary fibrosis and the differences between AD-114 and other CXCR4 antagonists such as AMD3100 suggests the interaction of AD-114 with CXCR4 is determined by greater complexity than binding kinetics alone. Finally, pirfenidone was also examined by us in our *in vivo* studies and by others, where this small molecule inhibitor was observed to reduce the Ashcroft score to a similar extent observed by AD-114, and to attenuate the circulating fibrocyte and macrophage pools^51^; however, these effects were only seen at a dose of 300 mg/kg/day. These observations suggest that progression of fibrosis is likely due to a complex interplay between CXCR4^+^ cells with different origins, including fibrocytes, epithelial cells, fibroblasts and immune cells, in the fibrotic lung. More research is warranted to better understand the role of CXCR4 signaling on these cells and in the overall fibrotic process.

While CXCR4 is thought to be a target in fibrotic lung diseases, targeting this chemokine receptor clinically has proved to be challenging. AMD3100 is registered for use in mobilizing stem cells from the bone marrow^52^, and has been used extensively in proof of concept studies to demonstrate that blocking CXCR4 in animal models of fibrosis could be a promising approach for anti-fibrotic therapy in humans^23,24,53,54^. However, AMD3100 has been shown to exert significant toxicity^55^, induce the mobilization of cells through the bone marrow and appears to have off target effects that would limit its use in IPF in humans. AD-114 has been shown to be a highly specific antagonist to CXCR4 and importantly, unlike AMD3100, does not mobilize stem cells from the bone marrow^27^. Thus, given the observed efficacy of AD-114 in ameliorating the fibrotic responses both *in vitro* and *in vivo*, especially after half-life extension via PASylation^33^, this biologic may represent an attractive therapeutic for IPF patients.

In summary, we have confirmed that CXCR4 expression is greater in the lung of IPF patients than in non-disease controls and we have shown that CXCR4 is expressed in airway and hyperplastic honeycomb-associated epithelial cells, interstitial cells and immune cells. The i-body CXCR4 antagonist, AD-114, reduces IPF lung fibroblast invasion as well as collagen 1 deposition. *In vivo*, this i-body decreases accumulation of fibrocytes (CXCR4^+^Col1^+^CD45^+^) and reduces the degree of lung injury and fibrosis in a murine bleomycin model of pulmonary fibrosis. Taken together our data suggests that AD-114 might present a promising approach for treatment of IPF in humans and future studies are underway to assess the precise cell types involved in propagating CXCR4 mediated profibrotic mechanisms in lung fibrosis.

## Materials and Methods

### Study approval

Institutional Review Boards both at Cedars-Sinai Medical Center and the University of Michigan, approved all experiments with primary human tissue. Informed consent was obtained from all patients prior to inclusion in the studies described herein. Human lung tissue used in immunohistochemical analysis was obtained from The Alfred Lung Fibrosis Biobank (supported by the NHMRC Centre of Research Excellence in Lung Fibrosis). Ethical approval for the biobank (#336-13) was obtained from The Alfred Hospital Ethics Committee in accordance with the National Statement on Ethical Conduct in Human Research.

### Cells and cell culture conditions

IPF diagnostic surgical lung biopsy samples were obtained from the NIH-funded Lung Tissue Research Consortium (www.ltrcpublic.com). Patients were followed for 6–12 months after diagnosis and were categorized as rapid or slow progressing IPF patients as previously defined^29^. Fibroblasts were generated from IPF diagnostic surgical lung biopsies or non-diseased control (NDC) non-fibrotic lung explants. Cells were grown in DMEM (Lonza) + 15% FBS (Cell Generation), 100 IU penicillin and 100 µg/mL streptomycin (Mediatech), 292 µg/mL L-Glutamine (Mediatech) and 100 µg/mL of Primocin (Invivogen) at 37 °C and 15% CO_2_.

### Chemical drugs

The small molecule drug AMD3100 (plerixafor, Mozabil) which is a specific inhibitor of CXCR4 approved for applications in stem cell mobilization^28^ was purchased from Tocris Bioscience as the octahydrochloride salt. BIBF-1120 (Nintedanib, OFEV®) is a multiple receptor tyrosine kinase inhibitor that is approved for IPF and was purchased from Cayman Chemical. Pirfenidone (Esbriet®) is currently approved for treatment of IPF and was purchased from Tocris Bioscience. Bleomycin was purchased from Eurasia Pharmaceuticals.

### Molecular Biology, protein purification and i-body validation

AD-114 i-bodies were expressed in *Escherichia coli* or *Pichia pastoris* with three different C-termini. The N-terminal CXCR4-reactive AD-114 i-body component was identical in all formats^27^. AD-114 and the negative i-body in “Im7-FH” format were tagged with the ~11.9 kDa Im7-FLAG-His_6_ fusion protein and were cloned, expressed and purified from *E. coli* as described by Griffiths *et al*.^27^. AD-114-6H tagged with His_6_ hexapeptide was cloned, expressed and purified from *P. pastoris* using Lonza’s XS expression technologies™. AD-114-6H was purified from the *P. pastoris* culture supernatant by immobilized metal affinity chromatography (HiPrep IMAC FF 16/10, GE Healthcare). AD-114-PA600-6H was expressed in *E. coli* as a fusion of AD-114 with a His_6_ hexapeptide-tagged ~47.8 kDa P/A#1 sequence comprising 600 residues^33^. Met-Ala-AD-114-PA600-6H (see Fig. 1) was purified by immobilized metal affinity chromatography (His-trap HP column, GE Healthcare) followed by subtractive anion exchange (Source15Q column, GE Healthcare) and then size exclusion chromatography (HiLoad 26/60 Superdex S200, GE Healthcare). AD-114-PA600-6H with no additional N-terminal residues (see Figs 5 and 8) was purified by fractionated ammonium sulfate precipitation (at 950 mM (NH_4_)_2_SO_4_) followed by subtractive anion exchange chromatography (Source15Q column, GE Healthcare).

Kinetic binding analysis of all i-bodies was confirmed by surface plasmon resonance (SPR) as previously described by Griffiths *et al*.^27^. Briefly, using a BIACore T200 serial dilutions of i-bodies were injected over captured human or murine biotinylated CXCR4 lipoparticles (Integral Molecular). The CXCR4 lipoparticle surface was regenerated with 0.1 M citrate pH 5.0 for 15 sec at 30 µL/min.

### Pharmacokinetics studies

Murine pharmacokinetic studies were conducted at ITR Canada laboratories (Canada) in accordance with the principles outlined in the “Guide to the Care and Use of Experimental Animals” as published by the Canadian Council on Animal Care and the NIH’s “Guide for the Care and Use of Laboratory Animals”. The study was approved by the Animal Care Committee (ACC) of ITR Canada. Mice were housed individually in a controlled environment of 21 ± 3 °C, relative humidity 50 ± 20%, 12 h light, 12 h dark. Blood samples (0.3 mL) were taken via the saphenous vein or under isoflurane anesthesia by cardiac puncture using K_2_-EDTA as anticoagulant. Blood samples were taken from each mouse at 2 time points and 3 mice were sampled per time point. Following collection, samples were centrifuged at 4 °C and the resulting plasma was stored at −80 °C in Protein LoBind tubes (Eppendorf). Animals were euthanized by cervical dislocation and discarded without further examination. Pharmacokinetic analysis was performed using Phoenix® WinNonlin® 6.3, Phoenix® Connect™1.3.1 software.

#### Study 1, AD-114-Im7-FH

Male Crl:CD1 (ICR) mice (Charles River Canada Inc.) were injected intravenously (IV) with a single dose of AD-114-Im7-FH at 3 mg/kg (3 mL/kg) and blood samples were collected 5, 15, 45, 60 and 90 min post dosing. AD-114-Im7-FH was quantified in plasma by LC-MS/MS (Algorithme Pharma, Canada) by monitoring the signature peptide LTPNQQR. Twenty-five μL of thawed plasma was combined with 25 μL of 8 M urea and incubated at 60 °C for 30 min. Twenty-five μL of stable labelled (^13^C^15^N) internal standard (IS) peptide GEKLTPNQQR*IG (0. 25 μg/mL) was added, followed by 200 μL of trypsin (2 mg/mL in 100 mM NH_4_HCO_3_). The sample was vortexed briefly then incubated at 60 °C for 90 min. Fifty μL of 10% HCOOH was added followed by vortexing and then centrifugation at 14 000 rpm for 2 min, 175 μL of supernatant was then transferred to a clean tube ahead of LC-MS/MS using a AB Sciex QTRAP5500 triple quadrupole mass spectrometer. Reverse phase chromatography was performed over a XBridge BEH300 C18, 50 × 2.1 mm, 3.5 μm column at 60 °C with a flow rate of 600 μL/min using mobile phases A [0.1% (v/v) aqueous HCOOH] and B [0.1% (v/v) HCOOH in acetonitrile].

The MS instrument used a turbo spray ESI ion source in positive mode and the multiple reaction monitoring (MRM) as method.

#### Study 2, AD-114-PA600-6H

Male Crl:CD1 (ICR) mice (Charles River Canada Inc.) were injected IV with AD-114-PA600-6H at 10 mg/kg (3.1 mL/kg) and blood samples were collected at 15, 30 min, 1, 2, 3, 4, 8, 12, 18, 24, 36, 48 and 72 h post dosing. Plasma samples were diluted 1/10 in 1% (w/v) BSA (Sigma) in PBS and AD-114-PA600-6H was quantified by ELISA using anti-human NCAM-1 (R&D Systems) to capture i-body and anti-Histidine-HRP (BioRad) to detect His_6_ tagged i-body. Absorbance of the HRP reactive reagent 3,3´,5,5´-tetramethylbenzidine (TMB) was quantified at 450 nm using a Biotek Powerwave XS or Synergy HT microplate reader (Biotek).

### Immunohistochemistry

Human lung parenchymal tissue sections were fixed in 10% (v/v) aqueous neutral buffered formalin overnight and subsequently transferred into tissue cassettes and placed in 70% (v/v) aqueous ethanol. The tissues were then paraffin embedded. Slides containing 4 µm sections were deparaffinized and hydrated. Antigen retrieval was performed by incubating the slides in 10 mM citric acid solution (pH 6.0). Slides were then stained with a commercially validated anti-CXCR4 antibody (clone UMB2, Abcam) followed by anti-rabbit polyclonal detection antibody (Abcam) and the Dako EnVision anti-rabbit kit (Dako Corp.) with 3,3′-diaminobenzidine (DAB) (Dako Corp.); sections were then counterstained with hematoxylin. Slides were alternatively stained with His6 tagged i-bodies followed by staining with biotinylated anti-His tag antibodies (Miltenyi Biotech) and visualised using a horse radish peroxidase - DAB (HRP-DAB) cell and tissue staining kit according to the manufacturer’s instructions (R&D systems).

### SCID/bg mice

SCID/Bg mice were intravenously administered with CCRF-CEM cells as previously described^36^. Mice were sacrificed 25–27 days after cellular administration and their spleens and livers were fixed, paraffin embedded, sectioned and stained with AD-114-6H or control i-body.

### Analysis of human IPF explant cells

#### Cell isolation

Lung explanted tissue was obtained from IPF patients at Cedars-Sinai Medical Center (Los Angeles, CA). Rejected donor lungs from non-diseased individuals were utilized as non-donor controls. Normal and IPF lung explants were placed into sterile PBS, washed and transferred into fresh PBS. Tissue was minced and centrifuged at 600 × g for 5 min. Supernatants were collected with the PBS utilized to wash the explanted lungs (lung wash). The top layer of the pellet enriched in mechanically dissociated cells and red blood cells (RBCs) were strained through a 70-µm strainer, the strainer washed several times with DPBS to separate cells from the minced tissues. This procedure was repeated until the RBCs and dissociated cells were removed from the minced tissue pellet. The dissociated cells were mixed with the lung wash and spun down at 400 × g for 5 min. RBCs were lysed using RBC lysis buffer (Biolegend) and cells were then counted and viably frozen down using CryoStor CS10 freezing medium (STEMCELL Technologies Inc.). The IPF lung biopsies were classified as slow or rapid-IPF based on the rate of FVC and/or DLCO decline, acute exacerbations or mortality, as previously described^29^.

#### Flow cytometry

Lung explant cells from NDCs or IPF patients were washed and resuspended at 1 × 10^7^ cells/mL in flow cytometry staining buffer (DPBS + 1% BSA + 0.02% NaN_3_). One hundred µL containing approximately 1 × 10^6^ cells were blocked for 15 min on ice using 2 µg of non-immune human IgG. Fluorescent conjugated CXCR4 and CD73 or isotype control antibodies (Biolegend) were added to the cells at a dilution of 1:50. The cells were incubated for 15 min on ice in the dark and subsequently washed twice with flow buffer and fixed in 5% neutral buffered formalin (NBF). A MACSQuant 10 (Miltenyi Biotech) flow cytometer was utilized for flow cytometric analysis and data were analyzed using FlowJo software (Treestar Inc.).

#### Invasion assay

ImageLock 96 well plates (Essen Bioscience) were coated with 50 µg/mL of basement membrane extract (BME; Trevigen) for one hour at room temperature. Fibroblasts generated from NDCs or slow or rapid progressing IPF patients were plated (36, 000 cells per well) on the BME-coated plates and incubated overnight. The following day cells were scratched using a Woundmaker™ (Essen Bioscience), washed with DPBS and treated with i-bodies (0.17, 0.83, 1.66, 4.2 or 10 µM) or AMD3100 (12 µM) in a 2 mg/mL BME solution. The BME was allowed to polymerize by incubating the plate at 37 °C for 15 min. The plates were then inserted into an IncuCyte Zoom imaging system and images were acquired every 2 h for 40 or 50 h. Invasion was quantified using IncuCyte software (Essen Bioscience).

#### Collagen1 ELISA

Fibroblasts were plated into 12 well plates (125, 000 cells per well) in DMEM (Lonza) + 15% FBS (Cell Generation), 100 IU penicillin and 100 µg/ml streptomycin (Mediatech), 292 µg/ml L-Glutamine (Mediatech) and 100 µg/ml of Primocin (Invivogen) and incubated overnight at 37 °C and 10% CO_2_. The cells were then either untreated or treated with AD-114-PA600-6H at 0.17, 0.83, 1.66 and 4.2 µM, or with negative i-body at 4.2 µM. After 48 h, the conditioned supernatants were collected for ELISA analysis. Collagen 1 was detected using a direct ELISA. Briefly, serially diluted purified collagen 1 was utilized to generate a standard curve. Collagen 1 standard and conditioned fibroblast supernatants were coated on MaxiSorb ELISA plates overnight at 4 °C. The next morning, plates were washed, blocked with 1% BSA for 1 h and then incubated with a biotinylated anti-Collagen 1 antibody (Abcam) for 2 h on a rotating shaker at room temperature. Plates were then washed and HRP-conjugated streptavidin was added to the wells for 20 min. The plates were washed and developed with TMB liquid substrate for 20 min after which a stop solution was added and the absorbance was measured at 450 nm using a Synergy H1 microplate reader (Biotek).

#### qPCR analysis

Fibroblasts were plated on 50 µg/mL of BME and treated with 10 µM of i-bodies or 12 µM of AMD3100. After 48 h, RNA was extracted using Trizol reagent and reverse transcribed into cDNA using superscript II reverse transcriptase (Life Technologies) as previously described^29^. Complementary DNA (cDNA) was subsequently loaded into a Taqman plate and gene expression analysis were performed using predesigned primers for *ACTA2, COL1A1, COL3A1* and *FN1*. All Taqman analysis was performed using Applied Bio system’s Viia 7 instrument (Life Technologies). The results were then exported, normalized to 18S RNA expression and fold change analyses were calculated using Data Assist software (Life Technologies).

### Murine bleomycin model of lung injury

#### Prophylactic and therapeutic treatment models

All animal experiments were conducted according to MuriGenics’ (USA) Institutional Animal Care and Use Committee protocol MG-30, most recently approved March 2017, and approved by MuriGenics’ Institutional Animal Care and Use Committee (IACUC). C57/BL6 male mice (age, 8–10 weeks) were purchased from Charles River Laboratories. All animals were acclimated for 3 days prior to the start of the study, housed in microisolators in a 12:12 light dark cycle and fed standard maintenance rodent chow diet and tap water *ad libitum*. Mice were randomized by body weight and divided into treatment groups. Lung injury was experimentally induced at day 0 by intratracheal instillation of a single dose of BLM (2U/kg body weight in 50 µL sterile saline) whilst naïve control mice received 50 µL saline. Test item treatments were delivered intraperitoneally. Mice were anesthetized with an isoflurane/CO_2_ mixture. For the 4 day prophylactic mode study, mice (n = 10/group) were dosed 1 h prior to BLM instillation with vehicle (saline), negative i-body (30 mg/kg), AD-114-FH (1, 10 or 30 mg/kg), AMD3100 (10 mg/kg), or pirfenidone (30 mg/kg), and then dosed daily until day 4. For the 21 day prophylactic mode study, mice (N = 5/group) were dosed 1 h prior to BLM instillation with vehicle (PBS), negative i-body (10 mg/kg) or AD-114-Im7-FH (10 mg/kg) and were then dosed daily with these treatments until day 21. For the therapeutic mode study, BLM-treated mice (N = 5/group) were dosed with vehicle (10 mM Na-citrate/citric acid, 100 mM NaCl, pH 6.2), AD-114-PA600-6H (10 mg/kg) or AMD3100 (10 mg/kg) daily from days 8–21. A control group that was not BLM treated received vehicle (10 mM Na-citrate/citric acid, 100 mM NaCl, pH 6.2) daily from days 8–21. An additional BLM-treated control group received no test item treatments and was anaesthetized at day 8. Bronchoalveolar lavage (BAL) fluid was collected as described by Song *et al*.^24^ and CXCL12 ELISA was completed using DuoSet ELISA Development System (R&D Systems) as described by Song *et al*. and Phillips *et al*.^24,50^.

#### Murine lung primary cell isolation and flow cytometry

Procedure was performed at 4 °C. Dissected lungs were rinsed in ice cold Hank’s Balanced Salt Solution (HBSS), cut into small pieces and then treated with 5 mL digestion buffer [0.2% (w/v) collagenase A (Roche), 2.4 U/mL dispase (Roche), 2 mM CaCl_2_, 10 mM HEPES, 150 mM NaCl) at 37 °C for 45 min, with gentle tritiation every 5–10 min. The tissue and cell suspension was strained through a 70 µm filter, washed with 25 mL of PBS and then centrifuged at 400 × g for 5 min. The cell pellet was triple stained with CD45, CXCR4 and Col1a: 100 µL of lung primary cells at 1 × 10^6^ cells/mL were washed twice with staining buffer [PBS + 2% (v/v) fetal calf serum] and then fixed and permeabilised with 200 µL BD Cytofix/Cytoperm^TM^ solution (BD Biosciences) for 20 min at 4 °C. Cells were washed with 2 × 1 mL of BD Cytofix/Cytoperm^TM^ solution and liquid was removed by decanting before addition of 2 µL Fc block (eBioscience). Fc block was incubated with cells for 10 min at 4 °C, followed by addition of the triple-stain antibody cocktail containing 10 µL per 10^6^ cells of CD45-PerCP (R&D Systems), 0.5 µg per 10^6^ cells of anti-mouse CXCR4-PE (Biolegend) and 2 µL per 10^6^ cells of anti-collagen1 (Abcam). Antibodies were incubated for 30 min at 4 °C then washed with 1 mL of BD Cytofix/Cytoperm^TM^ solution. To detect the anti-collagen 1 antibody, 2.5 µL of anti-mouse 488 secondary antibody (Abcam) was then added followed by incubation for 30 min at room temperature. Stained cells were washed twice with 1 mL of BD Cytofix/Cytoperm^TM^ solution and resuspended in 100 µL of this buffer followed by flow cytometry analysis. A BD FACS Calibur flow cytometer was utilized for flow cytometric analysis and data were analyzed using Cell Quest software.

#### qPCR

All tissue samples were stored at −80 °C until preparation. RNA was extracted from lung tissue using the King Fisher system (Thermo Fisher Scientific) on the Biosprint machine (Qiagen) protocol was performed to the manufacturer’s specifications. Briefly, lung tissue (25 mg) was thawed and combined with Pure Viral lysis buffer (600 μL, King Fisher) with proteinase K (60 μL) in 96 deep-well grinding block with two steel beads per well. Samples were homogenised for 2.5 min at 1750 rpm using the Spex SamplePrep Geno/Grinder 2010 (Spex Sample prep). Homogenised samples were incubated at 56 °C for 25 min, lysate was extracted using the S-block machine. Final RNA extraction was performed using magnetic beads (25 μL, King Fisher), samples was washed with isopropanol and eluted with RNase free water. Concentration of RNA extracted was determined using a Nanodrop 2000 spectrophotometer (Thermo Fisher Scientific). cDNA preparation was done using a High-Capacity cDNA REVERSE Transcription kit (Applied Biosystems) according to the manufacturer’s instructions. The amount of input RNA for the reaction was 200 ng. The common reaction mixture was made for a total volume of 10 uL which included the input RNA and the PCR conditions were 25 °C for 10 min, 37 °C for 120 min, 85 °C for 5 min. The cDNA was stored at 4 °C. Each qPCR reaction contained 20× primers and probe, with a final concentration of 400 nM for each primer and 80 nM for the hydrolysis probe. All primers were Taqman primers from (Applied Biosystems). Primers used were beta actin (ActB, Mm02619580_g1), collagen 3a1 (Col3a1, Mm01254476_m1), collagen 1a1 (Col1a1, Mm00801666_g1) and alpha Smooth Muscle Actin (SMAα, Mm00725412_s1). In a final reaction volume of 12 μL: 6 μL of PCR master mix (TaqMan Gene Expression PCR Master Mix, Thermo Fisher Scientific) and 5 μL of cDNA and 1 μL primer probe. qPCR was performed using an automated fluorimeter (ABI PRISM 7900 HTA FAST, Thermo Fisher Scientific). The following cycling conditions were used: 2 min at 50 °C, 10 min at 95 °C, 40 cycles of 15 s at 95 °C and 60 s at 60 °C. Fluorescent signals were collected during the annealing phase and C_T_ values extracted with a threshold of 0.2 and baseline values of 3–10 for the genes of interest and 0.1, 5–10 for the reference gene, ActB. For each sample C_T_ values were calculated and normalized to the house keeping gene ActB, (Gene C_T_ − ActB C_T_ = ΔC_T_). ΔC_T_ values were then compared to the relative gene expression of a target to naïve mouse samples (ΔC_T_(Sample) − ΔC_T_(naïve) = ΔΔC_T_). % fold change was determined as 2^ΔΔC_T_ × 100. All values are expressed as mean ± S.E.M. Samples that returned a C_T_ value greater than controls (>30) were excluded from analysis.

#### Histology and Ashcroft scoring

Lungs were removed and fixed in Neutral Buffered Formalin (10%; Richard-Allan Scientific™; Thermo Fisher Scientific) for 48 h and then processed in a Leica TP 1020 tissue processor and embedded using a Leica EG 1140 H paraffin embedding module and embedding centre (Leica EG 1140 H; Leica Biosystems). The blocks were then sectioned into 2 serial sections per slide, at 5 μm each using a Leica RM 2155 microtome (Leica Biosystems). Slides were stained using the Gomori’s Trichrome Kit (Richard-Allan Scientific™; Thermo Fisher Scientific) as per the manufacturer’s instructions. Random fields were imaged with a Nikon Plan 20× objective on a Nikon E600 microscope equipped with a Canon 70D EOS digital camera using EOS Utility on a MacBook Air. RAW images were converted to TIFF files in Photoshop CS6. Ashcroft scores were determined by measuring the severity of patterns of interstitial fibrosis (including thickening of alveolar or bronchiolar walls, damage to lung structure, formation of fibrous masses, honeycomb cysts) according to the method of Ashcroft *et al*.^37^. For each animal the mean score from 15 fields was determined and then averaged across the number of animals per group and presented as mean ± S.E. Differences were considered statistically significant if *p* values were less than 0.05.

### RNA Seq Assessments

A minimum of 700 ng of total RNA was used in “Dynabeads® mRNA DIRECT™ Micro Purification Kit” (Ambion). The libraries were prepared using the Ion Total RNA-Seq Kit v2 (Life Technologies). Samples were then loaded into an Ion Torrent for amplification onto Ion Sphere Particles using Ion PI™ Template OT2 200 Kit v3 (Life Technologies), sequencing chemistry, Ion PI™ Sequencing 200 Kit v3 (Life Technologies) and sequencing Chip, Ion PI™ Chip Kit v2 (Life Technologies). Samples were sequenced to at least 10 million reads. Raw reads in FASTQ format were aligned to the UCSC human reference genome (hg19) using TOPHAT. Gene expression was calculated using a gene transfer file (GTF) from UCSC genes and normalized read quantification as FPKM (Fragments Per Kilobase of transcript per Million fragments mapped) calculated with Cufflinks. Average FPMK values for various senescence associated markers were mined and are depicted in Figure S6.

### Statistical analysis

All data were analysed using GraphPad Prism (version 7) for statistical significance by ordinary One-way or Two-way ANOVA, followed by Tukey’s or Dunnett’s post-hoc test.

### Data availability

The datasets generated during and/or analysed during the current study are available from the corresponding author on reasonable request.

## Electronic supplementary material

Supplementary Data S1-S4
