# Supplementary material for: Anti-fibrotic Effects of CXCR4-Targeting i-body AD-114 in Preclinical Models of Pulmonary Fibrosis
Source: Sci Rep. 2018 Feb 16;8:3212. doi: 10.1038/s41598-018-20811-5 (PMC5816662; doi:10.1038/s41598-018-20811-5)
Supplement: Supplementary file 1 — Supplementary Data S1-S4 [file 41598_2018_20811_MOESM1_ESM.docx]

**Anti-fibrotic Effects of CXCR4-Targeting I-body AD-114 in Preclinical Models of Pulmonary Fibrosis**

*K. Griffiths^1‡^, D. M. Habiel^2‡^, J. Jaffar^3^, U. Binder^4^, W. G. Darby^1^, C. G. Hosking^1^, A. Skerra^4^, G. Westall^3^, C. M. Hogaboam^2^ and M. Foley^1*^*

*^1^AdAlta Limited, 15/2 Park Drive Bundoora, Australia 3083 and La Trobe University, Department of Biochemistry and Genetics, La Trobe University, Bundoora, Melbourne, Australia, 3086*

*^2^Cedars-Sinai, Los Angeles, CA, USA 90048*

*^3^Department of Allergy, Immunology and Respiratory Medicine, Monash University, The Alfred Hospital, Melbourne, Victoria, Australia 3000*

*^4^ XL-protein GmbH, Lise-Meitner-Str. 30, 85354 Freising, Germany*

*^‡^* Contributed equally to this work.

^*^ Correspondence to [M.Foley@latrobe.edu.au](mailto:M.Foley@latrobe.edu.au)

CXCR4


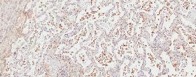

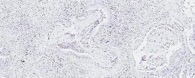

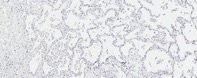

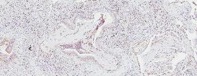

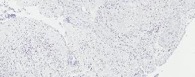

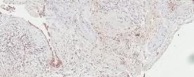

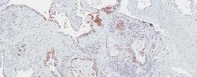

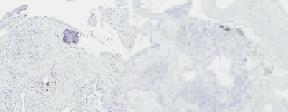

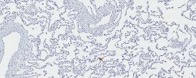

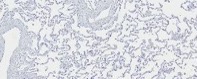

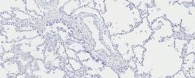

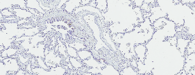

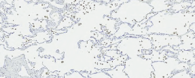

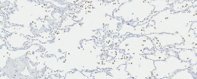

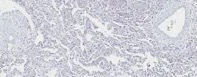

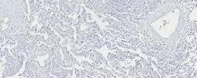


IPF

NDC

Secondary alone

AS

AS

BV

BV

BV

EP

EP

BV

HC

HC

MP

FF

TI

**Figure S1: Immunohistochemical staining of human lung tissue from 4 patients with IPF (top panels) and 4 non-diseased control (NDC) donors without lung disease (bottom panels).**

CXCR4 positivity was identified using commercially available, validated antibody and visualized using DAB (brown). Tissue was counterstained in Mayer’s hematoxylin. Right side panels show staining with primary antibody omitted. Scale bars: 500 μm. AS alveolar space, BV blood vessel, EP epithelium, FF fibroblastic foci, HC honeycombing, MC mucus plug, TI thickened interstitium, SA small airway

**
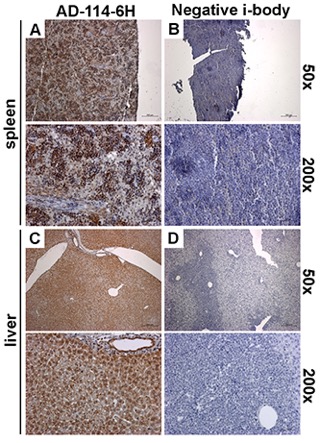
**

**Figure S2: Detection of CXCR4 expressing human leukemia T-Cells and mouse hepatocytes using AD-114-6H.**

SCID/bg mice were intravenously injected with CCRF-CEM cells and sacrificed after 27 days. Spleens and livers were collected and histologically analyzed. **(A-D)** Depicted are images of spleens (**A-B**) and livers (**C-D**) stained with AD-114-6H (**A, C**, brown staining) and negative i-body (**B, D**). Shown are images acquired at 50× (top) and 200× (bottom) magnification. Scale bars are 200 μm for 50× magnifications and 50 μm for 200× magnifications.

**Figure S3: AD-114-6H markedly reduced IPF lung fibroblast invasion.**

Lung fibroblasts were plated onto BME coated wells, scratched and then layered with 2 mg/ mL BME containing AD-114-6H and negative i-body at 10 μM or AMD3100 at 12 μM. Invasion of fibroblasts from NDC donors NL-A (**A**), NL26789 (**B**) and IPF patients IPF015 (**C**), S98 (**D**) and S175 (**E**) was measured for 50 h following treatment. N = 3, error bars show S.E. Statistical significance comparing the mean of AD-114 and the mean of AMD3100 is shown as *p = <0.05, **p = <0.01 as determined following testing using a two way ANOVA, Tukey’s posthoc test.


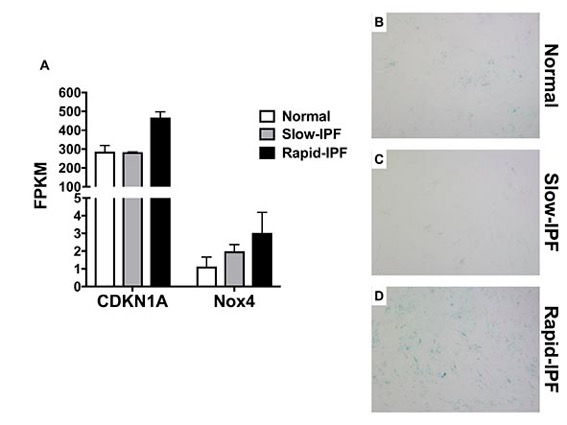


**Figure S4: Enrichment of senescence associated markers in IPF lung fibroblasts derived from progressive IPF patients.**

**(A)** RNA was extracted from normal, slow- and rapid-IPF fibroblast cultures and subjected to RNAseq analysis. Shown is the average normalized FPKM values for *CDKN1A* and *NOX4* transcripts. **(B-D)** Lung fibroblasts were cultured, fixed and β-galactosidase activity (blue) was determined using a β-galactosidase staining kit (Biovision).
